# On the Diminution and Disappearance of Uterine Tumors

**Published:** 1854-04

**Authors:** Samuel Ashwell

**Affiliations:** Late Obstetric Physician and Lecturer at Guy’s Hospital


*On the Diminution and Disappearance of Uterine Tumors.* By Samuel Ashwell, M. D., late Obstetric Physician and Lecturer at Guy's Hospital.

There is something so singular and unexpected in the *almost spontaneous* diminution and disappearance of large and hard tumors of the uterus, without coincident and appreciable breaking-down of their structure, that I think it may not be without advantage to bring together some of these unusual and striking cases, accompanying them with a few explanatory and practical observations.

Formerly, more than at present, the recognition of a large tumor in the pelvic region excited the alarm, not only of the patient, but of her professional attendant; and it is only from more accurate and extended pathological knowledge as to the indolent and innocuous character of some of these growths that we now regard them with less anxiety.

The exact pathology, however, of certain of these uterine tumors is still a vexed question—one certainly admitting of further elucidation—whether, for instance, a *fibrous* tumor is ever capable of being absorbed, is yet a matter of dispute; for while Lisfranc regards the absorption of any tumor as satisfactory proof that it is not fibrous, there are, as I shall show, distinguished pathologists, both in this country and abroad, who believe they have witnessed the melting down and disappearance of both fibrous and cancerous growths. It is therefore right, where we can only form a doubtful diagnosis as to the nature of any uterine tumor, to treat it as though it were curable; for if malignant, judicious treatment can do no harm; whereas, if it be of unspecific and benign character, the treatment will generally prove curative. I state, then, at once, that the pathology of these large and hard tumors, to the very occasional diminution and disappearance of which, *without* coincident breaking-down and discharge of their structure, I am now soliciting attention, is not the object of this paper, my aim being, to show that tumors of the uterus so large as to attract attention by their great size, so hard as to resemble, in some examples, the hardness of true carcinoma, and giving rise to alarming hæmorrhages and frequently-recurring pain, do very occasionally, when all treatment has been abandoned, present the following results:—

First. A slow but progressive diminution of bulk and of hardness, unaccompanied by any appreciable breaking-down of their structure.

Second. A lessening at least, and sometimes a cessation, of the bleedings, profuse menstruation and discharges, and, where it has existed, of the pain.

Third. A gathering of flesh, and a restoration of the general health.

Prior to the detail of the appended cases, it is necessary to make the following observations.

That, in all of them, treatment (especially by iodine, in tincture and ointment) had been long pursued; that nutritions, unstimulating diet, mild malt liquor, and light wines, were allowed; that resort was only occasionally had to *leeching* near, not over, the seat of pain; and still more rarely to cupping on the loins; that *purgatives* and aperients were exhibited only when it was evident that the bowels required to be unloaded; that *setons* over the site of the tumor produced, in several instances, marked benefit; and further, that in all the numerous cases which have fallen under my notice, the *recumbent posture*, and, as far as possible, the avoidance of sexual intercourse, but particularly the former, have been *strictly* enjoined.

*Large hard tumor of the uterus, with excessive hæmorrhages, and slow but complete diminution of bulk.*

Case 1.—April 22d, 1843.—Miss ——, aged forty-eight, resides near Hounslow, and has formerly been under the care of Dr. Blundell and her own medical attendant. First perceived a tumor about the size of a small melon three years ago. It was then low down in the hypogastric region. Her health did not then suffer, but two years since menstruation became profuse, and there was also much uterine bleeding. Iodine was used, and various means were employed to arrest the hæmorrhages.

Now the tumor is as large as a moderate sized adult head, lobulated, and in several of its more prominent portions of *extreme* hardness. It reaches nearly as high as the umbilicus, and protrudes the abdominal integuments, giving to the patient the appearance of a pregnancy of the fifth or sixth month. Has frequent cutting pains in and about the tumor, and is greatly inconvenienced by the weight, pressure, and tension. The growth is not tender to the touch, not even in those portions where there is constant pain. Walking is difficult. Internally the vagina is capacious, and there is much mucous discharge. The os uteri is patulous; and its lips, together with the cervix, are soft and swollen, but without any spots of induration. The most alarming symptom is the hæmorrhage; which, without any assignable cause, is sometimes so excessive as to induce long-continued faintness. Cold applications are often employed for several days before the bleedings are arrested. She has lost flesh, and is very weak; her countenance is anxious and very pallid; pulse 110; bowels constipated; appetite bad; she is restless and irritable, and often extremely depressed. Tincture of iodine, six minims, three times a day, in a little sugared water, and the iodine ointment every night over the tumor, were prescribed.

For several years I watched this patient, she being often in extreme danger from the bleedings. The use of the iodine was frequently suspended, and various remedies, rendered necessary by exhaustion, were employed in its stead. In 1847, Miss ——, being then fifty-two years of age, menstruation ceased, and at that period the hæmorrhages became far less frequent, and the tumor was manifestly less.

Nov. 22d, 1849.—Miss ——, called upon me, saying that the bleedings had returned very rarely, and never to great extent. She had regained her flesh, and was in very tolerable health. The tumor was not larger than an orange. By examination "per vaginam," I could discover scarcely any hardness of the cervix.

May 9th, 1851.—I find the following entry in my case-book:—"Today Miss —— calls to consult me about some slight derangement of her general health. I can, externally, scarcely make out any tumor, and as to the os and cervix, there is scarcely any appreciable duration."

March 22d, 1852.—Again Miss —— comes to me on account of slight indisposition. The tumor cannot be felt externally, and it is only by pressing the fingers deep down into the pelvis, behind the pubis, that it is at all perceptible. There has not been any vaginal discharge for several years. I may, in concluding the history of this case, remark, that I have very recently seen Miss ——, and, but for indigestion, she is in good health. There is just as much of the tumor to be felt as at the last examination.

[We omit the other cases related.]

*Remarks.*—I could narrate several more examples of nearly entire disappearance of these large tumors, and a still greater number where after having diminished very considerably in bulk, they have, for many years, remained entirely stationary. In both classes of cases the attendant symptoms have passed away, and there has been little or no interference with the comfort of life. All the cases which have fallen under my care prove the value, where any doubt exists, of the precise character of uterine growths, of cautious and long-protracted treatment. The continuance of pain, hæmorrhage, and emaciation, with a stationary condition of the tumor, although unfavorable, ought not to arrest the treatment; while, on the contrary, any diminution of these painful accompaniments should be regarded as sufficiently auspicious to encourage a further continuance of remedies. In *every* similar case the means recommended may not be attended with like success; still, in by far the greater number, I am strongly of opinion their employment will be decidedly efficacious, if not entirely curative. We are not expected to determine what may be the final issue of many uterine tumors, nor to deny the possibility of eventual malignant development, nor to promise a cure; but, judging from the narrated cases, and many others which I am sure have occurred to different practitioners, I may truthfully declare, that in many such thus treated, entire disappearance, or, at least, an arrest of further growth will be the result.—*London Lancet.*

